# Precise Position Control of Holonomic Inchworm Robot Using Four Optical Encoders

**DOI:** 10.3390/mi14020375

**Published:** 2023-02-02

**Authors:** Kengo Tanabe, Masato Shiota, Eiji Kusui, Yohei Iida, Hazumu Kusama, Ryosuke Kinoshita, Yohei Tsukui, Rintaro Minegishi, Yuta Sunohara, Ohmi Fuchiwaki

**Affiliations:** Department of Mechanical Engineering, Yokohama National University, 79-5 Tokiwadai, Hodogaya-ku, Yokohama 2408051, Kanagawa, Japan

**Keywords:** XYθ position control, holonomic inchworm robot, optical encoder, closed-loop control, calibration, crosstalk error

## Abstract

In this study, an XYθ position sensor is designed/proposed to realize the precise control of the XYθ position of a holonomic inchworm robot in the centimeter to submicrometer range using four optical encoders. The sensor was designed to be sufficiently compact for mounting on a centimeter-sized robot for closed-loop control. To simultaneously measure the XYθ displacements, we designed an integrated two-degrees-of-freedom scale for the four encoders. We also derived a calibration equation to decrease the crosstalk errors among the XYθ axes. To investigate the feasibility of this approach, we placed the scale as a measurement target for a holonomic robot. We demonstrated closed-loop sequence control of a star-shaped trajectory for multiple-step motion in the centimeter to micrometer range. We also demonstrated simultaneous three-axis proportional–integral–derivative control for one-step motion in the micrometer to sub-micrometer range. The close-up trajectories were examined to determine the detailed behavior with sub-micrometer and sub-millidegree resolutions in the MHz measurement cycle. This study is an important step toward wide-range flexible control of precise holonomic robots for various applications in which multiple tools work precisely within the limited space of instruments and microscopes.

## 1. Introduction

In recent years, electronic devices and their components have been miniaturized in microelectromechanical systems (MEMS) and mobile computers [1,2,3]. In biology, micromanipulation is necessary for delicate and fragile objects, such as biological cells, microfossils, and microorganisms [4,5,6,7].

Micromanipulation requires tools to accurately manipulate objects and multi-axis positioners to move the tool to an appropriate position and orientation with a sub-micrometer resolution. Various tools have been developed, such as vacuum nozzles [8], microtweezers with force sensors [4,9], and grippers that use capillary force [10,11]. Regarding the multi-axis positioner, the combination of a single-axis stage driven by linear synchronous motors with linear guides and stepping motors with ball-screw mechanisms are widely used in the industry [12,13]. These machines satisfy the requirements for micromanipulation in terms of accuracy, payload, and durability. However, they are considerably larger and heavier than the tiny parts. To adapt to a wider range of applications, multiaxis positioning stages should be sufficiently compact to use multiple tools simultaneously within the limited space of various instruments and microscopes.

Micromanipulation using small self-propelled robots has been studied to circumvent these problems [14,15,16,17,18]. This type of robot has three-degree-of-freedom (3-DoF) movements and is capable of holonomic movements. Therefore, it is expected to be used for monotonous XY-axis movement and complicated tasks, such as revolution around a tooltip within a narrow microscopic image.

**Figure 1:**
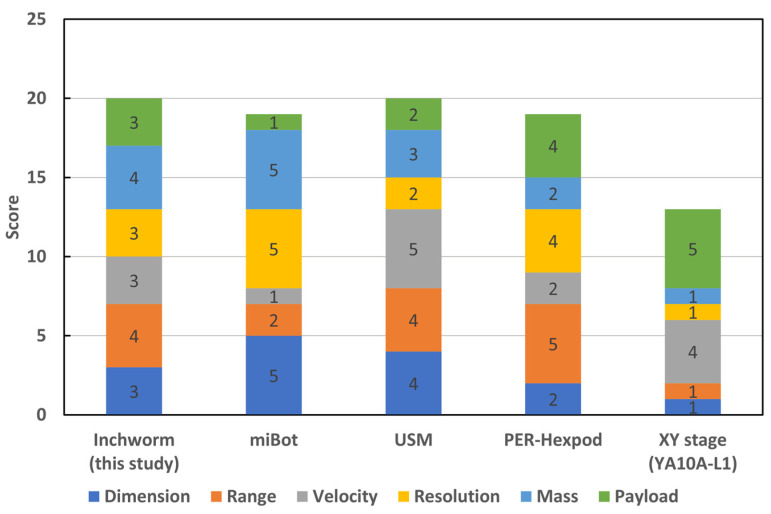
Comparison of typical micromanipulation robots and XY stage [22,24,27]. Scores are evaluated based on the reference [27] (refer to Table S1 of the Supplementary Materials for the quantitative comparison of the performances).

Various types of self-propelled robots have employed different principles and structures. Typical types of motion principles include stick–slip [19,20,21,22], centrifugal force [23], USM [24,25], and inchworm [26]. Figure 1 shows a comparison of the representative micromanipulation robots and the XY stage [22,24,27]. These robots have distinct advantages in terms of their specific performance, such as motion range, velocity, carrying capacity, and motion resolution. The performance of the inchworm robot was above average. Therefore, this robot can perform various micromanipulation tasks.

Previous research has also confirmed that the holonomic inchworm robots that we developed can be driven omnidirectionally under open-loop control [28]. However, self-propelled robots are susceptible to external disturbances, such as unevenness in the worktable and tension from the feeding wires. Therefore, precise and fast measurement of XYθ axis displacements with the sub-micrometer resolution is important for determining the exact position and orientation of robots.

Various methods have been developed to measure displacement precisely [29]. One of the most commonly used methods is pattern matching using camera images; however, this method has practical problems concerning a tradeoff among the measuring resolution, range, and cycle [30,31]. Strain gauges [32], lasers [33], and encoders [34,35] have also been used.

Optical encoders are feasible as multiaxis and fast position sensors because of their short measurement cycle, long range, and high resolution. In recent years, encoders have become significantly miniaturized, allowing them to be mounted on small robots. We previously reported an outline of an XYθ displacement sensor using four optical encoders, although we omitted the evaluation of the measurement performance [28,36]. In this study, we describe the evaluation and demonstration of the precise position control of a holonomic inchworm robot in the centimeter to the sub-micrometer range.

The remainder of this paper is organized as follows. In Section 2, we describe the holonomic inchworm robot, and we evaluate the XYθ displacement sensor in Section 3. The closed-loop sequence control for a multiple-step motion in the centimeter to micrometer range and simultaneous three-axis proportional–integral–derivative (PID) control for a one-step motion in the micrometer to sub-micrometer range are presented in Section 4 and Section 5, respectively. Finally, conclusions and future prospects are presented in Section 6.

## 2. Holonomic Inchworm Robot

### 2.1. Structure

**Figure 2:**
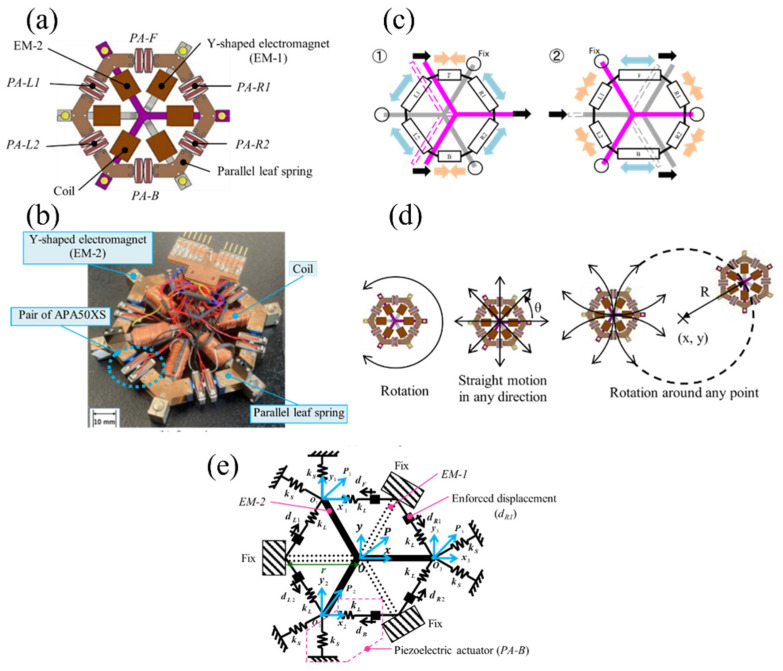
Holonomic inchworm robot: (**a**) structure (top view); (**b**) overview; (**c**) principle of motion (rightward movement); (**d**) motion patterns; (**e**) dynamic model.

Figure 2a shows the structure of the holonomic inchworm robot. *EM-1* and *EM-2* are pairs of Y-shaped electromagnets (EMs) that are separated to avoid mutual attraction. These EMs form a closed loop via a ferromagnetic surface to obtain a sufficient magnetic force to fix the floor. *PA-F*, *PA-B*, *PA-L1*, *PA-L2*, *PA-R1*, and *PA-R2* are piezoelectric actuators (PAs) with a mechanical displacement amplification mechanism. We arranged *EM-1* and *EM-2* to cross each other and connect them to the six PAs to move precisely in all directions according to the inchworm principle.

Figure 2b shows a photograph of the holonomic inchworm robot. The robot weighed 100 g and measured 86 mm × 86 mm × 11 mm. It had parallel leaf springs for the simultaneous smooth contact of all legs on the surface. Table 1 and Table 2 list the specifications of the robot and the PAs, respectively. We used an APA50XS “Moonie” PA (Cedrat Inc., Meylan, France) connected in series.

### 2.2. Principle

Figure 2c shows the motion sequence of the rightward orthogonal motions. It moves as an inchworm while retaining the synchronism between the rectangular forces of the two EMs and the vibrations of the six PAs. When the amplitudes of the vibrations are precisely controlled, it moves in any 3-DoF direction with a resolution of less than 10 nm.

As shown in Figure 2d, the robot can move linearly in any direction and rotate around any point with precision on well-polished ferromagnetic surfaces. If one EM is fixed to the floor, the other can be precisely positioned using six PAs.

### 2.3. Dynamic Model

As shown in Figure 2e, we defined a 3-DoF dynamic model of the robot when *EM-2* was free, and *EM-1* was fixed. Here, *k_L_* and *k_S_* are the spring constants of the six PAs in the compression and shear deformations, respectively. The *d_F_* is the enforced displacement of *PZT-F*, and *d_B_*, *d_R_*_1_, *d_R_*_2_, *d_L_*_1_, and *d_L_*_2_ are similar.

*EM-1* and *EM-2* were connected by hinged joints at *P*_1_, *P*_2_, and *P*_3_. *O*_1_, *O*_2_, and *O*_3_ are the initial positions of *P*_1_, *P*_2_, and *P*_3_, respectively. Furthermore, *x*_1_ and *y*_1_ are the coordinates of *P*_1_; (*x*_2_, *y*_2_) and (*x*_3_, *y*_3_) similarly define *P*_2_ and *P*_3_, respectively. *P* is the center of gravity of *EM-1*. The position of *P* is represented by the orthogonal coordinate system used for *x* and *y*. *O* is the origin of *P*. We assume that EMs are rigid bodies.

The six PAs and 3-DoF motions were determined based on the positions of *P*_1_, *P*_2_, and *P*_3_. Therefore, the PAs move to the free leg; *m* is the mass of the EMs, *I* is the moment of inertia of the gravity centers of the EMs, and *r* is the distance between the center and end of the EMs. From Figure 1, Newton’s equations of motion for *EM-1* and the related parameters are represented by Equations (1)–(7).

(1) $$M\ddot{y}+K\;y=Ku$$

(2) $$M=\left[\begin{array}{ccc} m & 0 & 0\\ 0 & m & 0\\ 0 & 0 & I/r^{2} \end{array}\right]$$

(3) $$y=\left[\begin{array}{c} x\\ y\\ r\theta \end{array}\right]$$

(4) $$u\equiv \left[\begin{array}{c} u_{x}\\ u_{y}\\ ru_{\theta } \end{array}\right]=K^{-1}K_{d}\;d$$

(5) $$K=\left[\begin{array}{ccc} 3\left(k_{L}+k_{S}\right) & 0 & 0\\ 0 & 3\left(k_{L}+k_{S}\right) & 0\\ 0 & 0 & 3\left(3k_{L}+k_{S}\right)/2 \end{array}\right]$$

(6) $$K_{d}=k_{L}\left[\begin{array}{cccccc} -1 & -1 & 1/2 & 1/2 & 1/2 & 1/2\\ 0 & 0 & \sqrt{3}/2 & -\sqrt{3}/2 & -\sqrt{3}/2 & \sqrt{3}/2\\ \sqrt{3}/2 & -\sqrt{3}/2 & -\sqrt{3}/2 & \sqrt{3}/2 & -\sqrt{3}/2 & \sqrt{3}/2 \end{array}\right]$$

(7) $$d={\left[\begin{array}{cccccc} d_{F} & d_{B} & d_{L1} & d_{L2} & d_{R1} & d_{R2} \end{array}\right]}^{t}.$$

(8) $$V=C\cdot \sin\omega t\left[\begin{array}{c} -W\cos\phi /3K_{1}+r\Theta /\sqrt{3}K_{2}\\ -W\cos\phi /3K_{1}-r\Theta /\sqrt{3}K_{2}\\ W\left(\cos\phi +\sqrt{3}\sin\phi \right)/2K_{1}-r\Theta /\sqrt{3}K_{2}\\ W\left(\cos\phi -\sqrt{3}\sin\phi \right)/2K_{1}+r\Theta /\sqrt{3}K_{2}\\ W\left(\cos\phi -\sqrt{3}\sin\phi \right)/2K_{1}-r\Theta /\sqrt{3}K_{2}\\ W\left(\cos\phi +\sqrt{3}\sin\phi \right)/2K_{1}+r\Theta /\sqrt{3}K_{2} \end{array}\right]+V_{0}\left[\begin{array}{c} 1\\ 1\\ 1\\ 1\\ 1\\ 1 \end{array}\right],$$

(9) $$V={\left[\begin{array}{cccccc} V_{F} & V_{B} & V_{L1} & V_{L2} & V_{R1} & V_{R2} \end{array}\right]}^{t}.$$

(10) $$\left[\begin{array}{c} K_{1}\\ K_{2} \end{array}\right]=\left[\begin{array}{c} k_{L}/\left\{3\left(k_{S}+k_{L}\right)-m\omega^{2}\right\}\\ k_{L}/\left\{3\left(3k_{L}+k_{S}\right)/2-I\omega^{2}/r^{2}\right\} \end{array}\right]$$

### 2.4. Input Voltages

Simplified voltages to the PAs for the translational motion were reported in [26]. In Equation (9), *V_F_*–*V_R_*_2_ are the input voltages to the PAs. *V_F_*–*V_R_*_2_ was obtained by solving Equation (1) using the approximation of harmonic oscillations with no damping.

Here, the moving direction is defined as ϕ, stride length during a half-step motion is *W*, half-orientation change of one step is Θ, angular frequency of the inchworm motion is ω, and the approximate proportional coefficient between the enforced displacements and voltage is *C*. A sinusoidal voltage was applied to the PAs up to 120 V_p-p_. The offset voltage was determined to be *V*_0_ = 60 V, which is half the maximum voltage of 120 V. *K*_1_ and *K*_2_ are given by Equation (10).

## 3. XYθ Position Sensor

### 3.1. Structure

**Figure 3:**
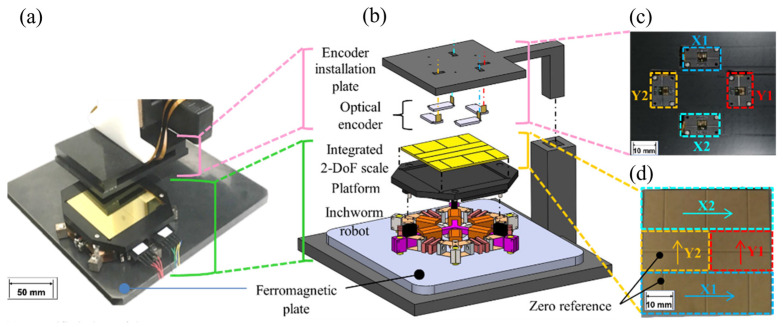
XYθ position sensor: (**a**) magnified view of the measurement area; (**b**) assembly drawing; (**c**) encoder installation plate; (**d**) integrated 2-DoF scale.

Figure 3 shows the proposed XYθ position sensor. Figure 3a shows a magnified view of the measurement area, and Figure 3b shows the assembly of the measurement components. As discussed previously, the robot moved on a ferromagnetic plate.

Four linear optical encoders (TA-200, Technohands, Yokohama Kanagawa, Japan), each with a resolution of 0.1 µm and a maximum measurement speed of 800 mm/s, were installed on the encoder installation plate, as shown in Figure 3c. We fixed the encoder installation plate above the robot to measure XYθ displacements in a stationary coordinate system.

The integrated 2-DoF scale was placed as a measurement target on top of one of the two legs, as shown in Figure 3d. The encoder specifications are listed in Table 3. The measurement range along the X–Y axis was 16 mm × 16 mm.

### 3.2. Signal Processing

**Figure 4:**
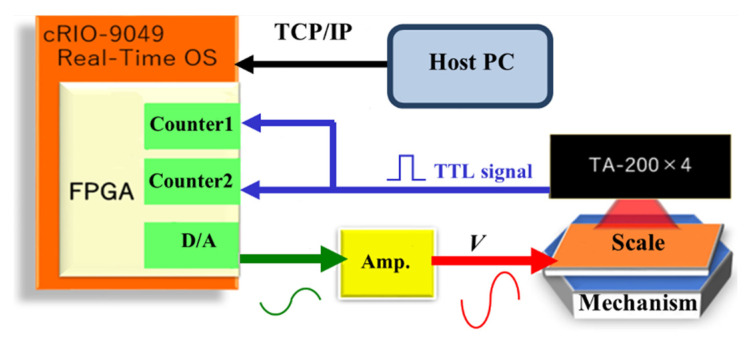
Signal processing.

As shown in Figure 4, we used a field-programmable gate array (FPGA) (C-RIO-9049, NI, TX, USA) to generate the control voltages of the robot and convert the transistor-transistor logic (TTL) signals from the encoders to XYθ displacements. The control voltages were magnified by 30 times using an amplifier circuit. The magnified signals are the input voltages to the PAs (***V***), as shown in Equation (9). The minimum measurement cycle of the FPGA was 0.35 μs, and the maximum calculated speed was 2800 mm/s. The FPGA measured the displacement of a sinusoidal vibration up to 1273 Hz with a displacement amplitude of 30 µm.

We assumed that the measurement cycle was sufficient because we moved the robot with an inchworm frequency of 100 Hz using sinusoidal displacement with an amplitude of 30 µm and a mechanical resonance frequency of less than 500 Hz.

Table 4 lists the performances of the position sensors. The measurement resolution was 0.1 μm with an uncertainty of ±0.2 μm for the X and Y axes in the static state. The measurement resolution was 0.3 millidegrees with an uncertainty of ±0.6 millidegrees for the θ-axis.

### 3.3. Measurement Principle

**Figure 5:**
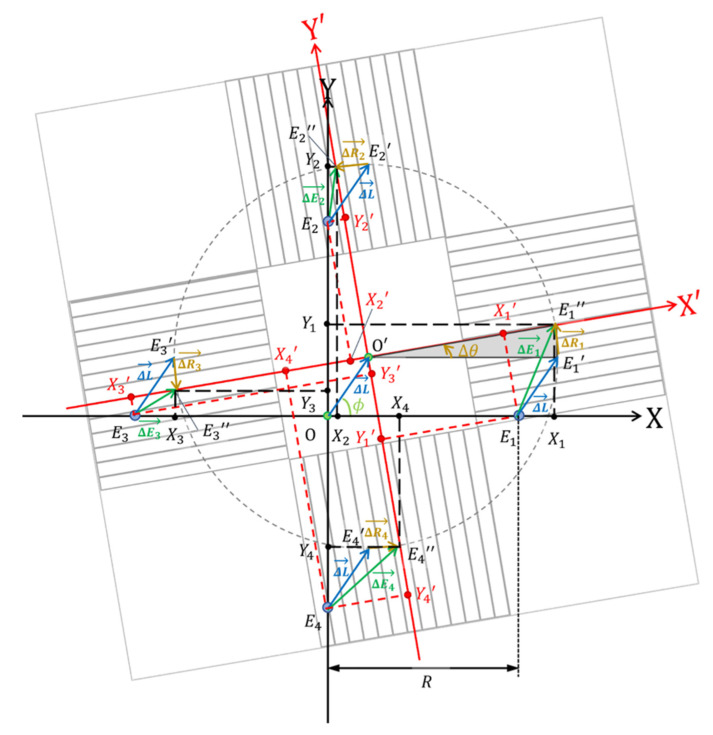
Vector diagram when the scale moves.

In this section, the conversion of the four measured distances into a 3-DoF motion of the scale is explained, and the major equations are presented. As shown in Figure 5, we performed vector analysis when the encoder installation plate was fixed to the coordinate system at rest and the scale was moved by the PAs, as shown in Figure 3.

We defined the *XY* and *XʹYʹ* coordinate systems as the coordinate systems at rest and on the scale, respectively. The coordinate transformation between *XY* and *XʹYʹ* is given as follows:

(11) $$\left[\begin{array}{c} X\\ Y \end{array}\right]=\left[\begin{array}{cc} \cos\Delta \theta & -\sin\Delta \theta \\ \sin\Delta \theta & \cos\Delta \theta \end{array}\right]\left[\begin{array}{c} X'\\ Y' \end{array}\right]+W\left[\begin{array}{c} \cos\phi \\ \sin\phi \end{array}\right].$$

where *E*_1_′ is the position after moving $\Delta \vec{L}$ from its initial position. $\Delta \vec{L}$ is defined as a vector component of the translational movement as follows:

(12) $$\Delta \vec{L}\equiv W\left[\begin{array}{c} \cos\phi \\ \sin\phi \end{array}\right]\equiv \left[\begin{array}{c} \Delta X\\ \Delta Y \end{array}\right]$$

where Δθ is the posture angle displacement of the scale, W is the translational movement, ϕ is the translational direction, ΔX is the translational movement along the X-axis, and ΔY is the translational movement along the Y-axis.

The 3-DoF motion is divided into translational and rotational motions. *E*_1_ is the initial position of encoder-1, which is defined as *(X*_10_, *Y*_10_) in the *XY* coordinate system. *E*_2_, *E*_3_, and *E*_4_ are defined similarly. *R* is the geometrical center of *E*_1_, *E*_2_, *E*_3_, and *E*_4._

(13) $$\left[\begin{array}{cccc} X_{10} & X_{20} & X_{30} & X_{40}\\ Y_{10} & Y_{20} & Y_{30} & Y_{40} \end{array}\right]=\left[\begin{array}{cccc} R & 0 & -R & 0\\ 0 & R & 0 & -R \end{array}\right]$$

where *E*_1_″ is the position of encoder-1 after moving $\Delta \vec{R_{1}}$ from *E*_1_′. $\Delta \vec{R_{1}}$ is defined as the vector component of rotational movement. We define (*X*_1_, *Y*_1_) as the *XY* coordinates of *E*_1_″, and (*X*_1_′, *Y*_1_′) as the *X*′*Y*′ coordinates of *E*_1_. The other parameters are defined similarly. We obtain the following coordinate transformation of the initial positions of *E_k_* from Equations (11) and (12) as follows:

(14) [Xk0Yk0]=[cosΔθ−sinΔθsinΔθcosΔθ][Xk'Yk']+W[cosϕsinϕ].

The measurable parameters are *Y*_1_′, *X*_2_′, *Y*_3_′, and *X*_4_′ using the four encoders, which are represented by Equation (14) as follows:

(15) $$\{\begin{array}{c} Y_{1}^{'}=-R\sin\Delta \theta -W\sin\left(\phi -\Delta \theta \right)\\ X_{2}^{'}=R\sin\Delta \theta -W\cos\left(\phi -\Delta \theta \right)\\ Y_{3}^{'}=R\sin\Delta \theta -W\sin\left(\phi -\Delta \theta \right)\\ X_{4}^{'}=-R\sin\Delta \theta -W\cos\left(\phi -\Delta \theta \right) \end{array}$$

From Equation (15), the displacements are represented as follows:

(16) [ΔXΔY]=−[cosΔθ−sinΔθsinΔθcosΔθ][X2'+X4'2Y1'+Y3'2]

(17) Δθ˜=−12{sin−1(X4'−X2'2R)+sin−1(Y1'−Y3'2R)}

where $\tilde{\Delta \theta }$ is the best estimator of Δθ and is the average of two expressions. When $\tilde{\Delta \theta }\ll 1$, (ΔX, ΔY, $\tilde{\Delta \theta }$) are approximated as follows:

(18) [ΔXΔYΔθ˜]≅−[1−Δθ˜0Δθ˜10001][01201212012014R−14R−14R14R][Y1'X2'Y3'X4'].

### 3.4. Experimental Results

The measurement performance of the sensor was evaluated by comparing it with the machine coordinates of a conventional precise XYθ stage. The XYθ stage was composed of a ball-screw type precise XY stage (KOHZU, Kawasaki Kanagawa, Japan, YA10A-L1) and θ stage (KOHZU, Japan, RA04A-W01) with the repeatability of 0.5 μm and 0.002°, respectively.

**Figure 6:**
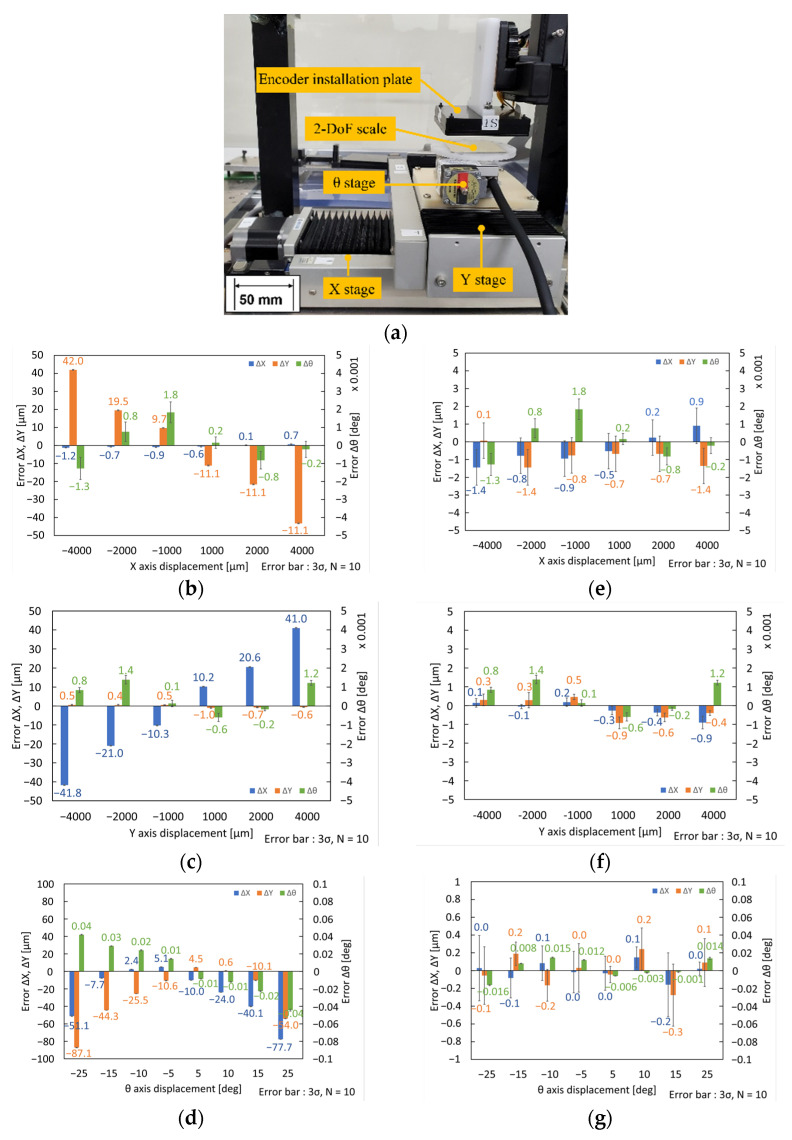
Measuring errors of XYθ-position sensor and variations in the ΔX, ΔY, and Δθ outputs after calibration: (**a**) Experimental setup for evaluation of the measuring performance of XYθ-position sensor; (**b**) Plots of XYθ-axes errors vs. X-axis displacement before calibration; (**c**) Plots of XYθ-axes errors vs. Y-axis displacement before calibration; (**d**) Plots of XYθ-axes errors vs. θ-axis displacement before calibration; (**e**) Plots of XYθ-axes errors vs. X-axis displacement after calibration; (**f**) Plots of XYθ-axes errors vs. Y-axis displacement after calibration; (**g**) Plots of XYθ-axes errors vs. θ-axis displacement after calibration.

Figure 6a shows the experimental setup. We replaced the robot with an XYθ stage, as shown in Figure 3. We fixed the encoder installation plate above the XYθ stage to measure the XYθ displacements from a stationary coordinate system. We placed the 2-DoF scale as the measurement target at the top of the θ stage.

Figure 6b shows the measurement errors of the XYθ-axes when the stage moves along the X-axis. Figure 6c,d show the errors in the XYθ-axes when the stage moves along the Y- and θ-axes, respectively. Nonlinear errors were caused by the XYθ-position sensor in the X- and Y-directions. The maximum error of the X-axis was approximately ±1.2 μm during the X-directional motion, and the maximum error of the Y-axis was approximately ±1.0 μm during the Y-directional motion. When the stage was moved along the θ-axis, the errors were closer to linear. The maximum error is approximately ±0.04° in the θ direction. The variations in the errors of the Y- and θ-axes are shown in Figure 6b; in other words, the crosstalk errors of the XYθ-position sensor. The maximum crosstalk errors were approximately ±40 μm and ±0.002° along the Y- and θ-axes. To reduce this crosstalk error, we calibrated the XYθ position sensors. We define the crosstalk error components as $C_{x}$, $C_{y}$, and $\theta_{0}$, where $C_{x}$ and $C_{y}$ are the eccentricities between the rotation axis of the θ stage and the geometrical center of the 2-DoF scale, $\theta_{0},$ is the posture angle of the misalignment between the axes of the sensor head and those of the 2-DoF scale. The displacements after calibration are expressed as follows:

(19) $$\left[\begin{array}{c} \Delta X_{c}\\ \Delta Y_{c}\\ \Delta \theta_{c} \end{array}\right]=\left[\begin{array}{ccc} \cos\theta_{0} & \sin\theta_{0} & 0\\ -\sin\theta_{0} & \cos\theta_{0} & 0\\ 0 & 0 & \frac{\sin\Delta \theta }{\sin\left(\Delta \theta +\theta_{0}\right)-\sin\theta_{0}} \end{array}\right]\left[\begin{array}{c} \Delta X\\ \Delta Y\\ \Delta \theta \end{array}\right]+\left[\begin{array}{c} -C_{x}(1-\cos\Delta \theta )-C_{y}\sin\Delta \theta \\ C_{x}\sin\Delta \theta -C_{y}(1-\cos\Delta \theta )\\ 0 \end{array}\right]$$

Figure 6e shows the measurement errors of the XYθ-axes after calibration when the stage was moved along the X-axis. Figure 6f,g shows those along the Y- and θ-axes, respectively. As shown in the figure, we performed a calibration using Equation (19), and the crosstalk errors significantly decreased in the X- and Y-directions. However, the crosstalk errors of the θ-axis in the X- and Y-directional motions did not improve significantly. The maximum residual crosstalk error of the X-directional movement was approximately 1.3 μm in the Y-direction, and that of the Y-directional movement was approximately 0.9 μm in the X-direction. In θ-directional motion, the maximum residual crosstalk errors were approximately 0.15 μm and 0.25 μm in the X- and Y-direction.

(See also Figure S1 for evaluating XYθ-axes errors for XYθ-axes simultaneous displacement for (X, Y, θ) = (4000 μm, 4000 μm, 5°)).

## 4. Sequence Control of Multiple Step Motions

### 4.1. Control Sequence

**Figure 7:**
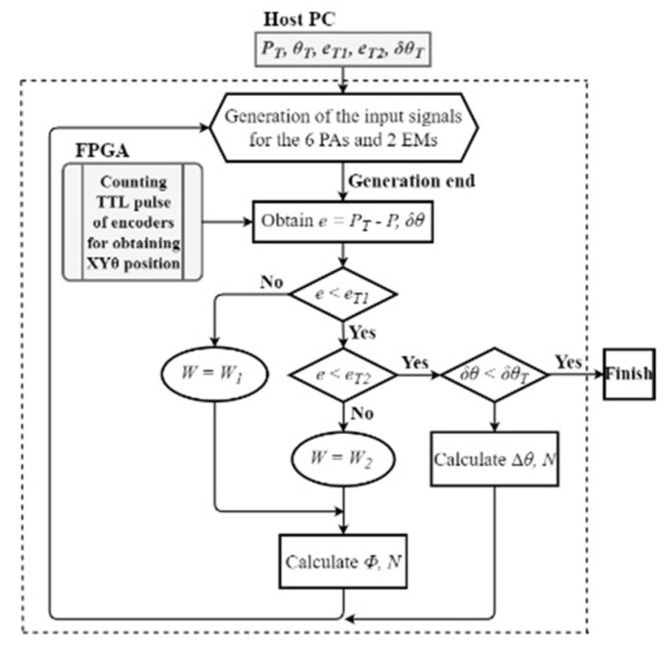
Sequence of navigation.

We demonstrated closed-loop control of star-shaped translational motion in the centimeter range. Figure 7 shows the control sequence that switches the combination of coarse and fine motions. Here, *P_T_* is the target position, and *e_T_*_1_ is the threshold of the distance error *e* for coarse motion; *e_T_*_2_ is the fine motion, *δθ_T_* is the threshold of the orientation error *δθ*, and *W*_1_ and *W*_2_ are the step lengths of the coarse and fine motions, respectively. We determined *e_T_*_1_ = 80 μm, *e_T_*_2_ = 15 μm, *δθ_T_* = 0.06°, *W*_1_ = 60.0 μm, and *W*_2_ = 5.0 μm. The robot moved to an inchworm frequency of 100 Hz.

### 4.2. Experimental Results

**Figure 8:**
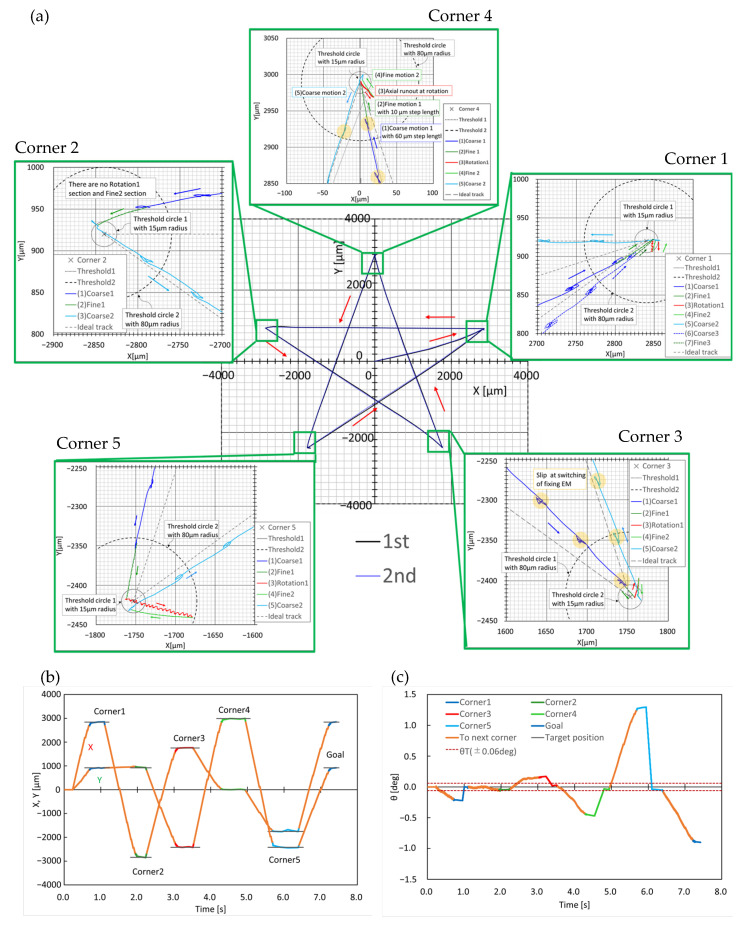
Star-shaped trajectories with five target points: (**a**) XY trajectory; (**b**) X, Y vs. time; (**c**) θ vs. time.

Based on the aforementioned conditions, the robot was controlled to draw a star shape with five corners as target points. The experiment was performed twice, and a map of the trajectories of the center of each robot is shown in Figure 8. As shown in Figure 8a, the first and second trajectories for each experiment almost overlapped; therefore, this mobile robot had high repeatability. In both trajectories, the average velocity was approximately 4.3 mm/s, whereas the velocity of the coarse motion was approximately 6.5 mm/s. In addition, close-up views around each corner of the first trajectory are shown. Regarding the trajectory, blue, green, and red indicate coarse, fine, and rotational motions, respectively. Considering corner four as an example, the robot first entered the range of *e_T_*_1_ with coarse movement, (1) Coarse1. It switched to fine movement and moved near the center of the range of *e_T_*_2_, (2) Fine1. If the center was within the range of *e_T_*_2_, the orientation angle *θ* was measured. If it was out of the range of *δθ_T_*, as shown in this case, the orientation was corrected by rotation, (3) Rotation1. If it moved outside the range of *e_T_*_2_, it approached the corner again using fine movements, (4) Fine2. If both the center and orientation were within the corresponding ranges of *e_T_*_2_ and *δθ_T_*, respectively, the robot changed to a coarse movement toward the next corner, (5) Coarse2.

As represented by the areas highlighted in yellow in Figure 8, cyclic vibrations occurred along the trajectory. We confirmed that these also appeared in the fine and rotational movements during every step by observing an enlarged view of the trajectories shown in Figure 8. Therefore, it is assumed that slippages of the electromagnetic legs occur during switching between the fixing and moving legs.

The plots of X, Y, and θ vs. time in Figure 8b,c show that the settling times around the corners were 0.2–0.8 s. We assume that the settling time can be reduced by improving the navigation sequence and motion compensation.

## 5. 3-Axis PID Control of One-Step Motion

### 5.1. Transfer Function

For precise positioning in the micrometer to sub-micrometer range, we demonstrated three-axis PID control of the one-step motion of *EM-2* when *EM-1* was fixed on the floor, as shown in Figure 1e. Considering the Laplace transform of Equation (1), we obtain the transfer function matrix P(s) as a second-order system:

(20) $$Y\left(s\right)\equiv {\left[s^{2}M+K\right]}^{-1}KU\left(s\right)$$

(21) $$P\left(s\right)={\left[s^{2}M+K\right]}^{-1}K$$

(22) $$U\left(s\right)=K^{-1}K_{d}\;D\left(s\right)$$

Here, we define the Laplace transform of ***y***(*t*) as ***Y***(*s*). The Laplace transforms of the other parameters are defined similarly.

**Figure 9:**
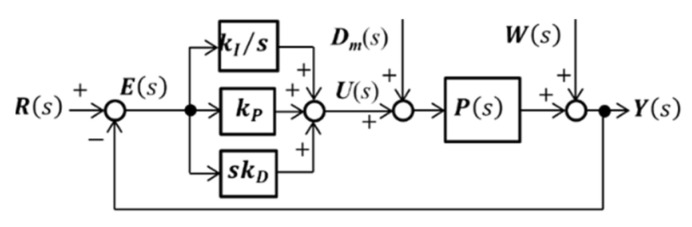
Block diagram of three-axe PID control.

Figure 9 shows a block diagram of the PID control. We define ***d_m_***(*t*) and ***w***(*t*) as vectors composed of the modeling error and uncertainties of the measured displacement, respectively. Here, ***k_I_***, ***k_P_***, and ***k_D_*** are vectors composed of integral, proportional, and derivative gains along the *x*-, *y*-, and *θ*-axes, respectively; ***r***(*t*) is the target position, and ***e***(*t*) is the deviation between ***r***(*t*) and ***y***(*t*). We applied a first-order Butterworth filter with a cutoff filter of 50 Hz to ***E***(***s***) before derivative (D) control to minimize the time delay of the primary experiments.

### 5.2. Experimental Results

In the experiments, we adjusted ***k_I_***, ***k_P_***, and ***k_D_*** for each experimental condition by using a heuristic method to obtain a no-overshoot trajectory. We conducted experiments five times for each condition, and similar results were obtained. We determined the target travel lengths *r_i_* as 1, 5, and 10 μm and the target moving directions *φ_i_* as 0°, 30°, 60°, and 90° for translational movements. We also determined the target rotational displacement of *θ_i_* as 0 and 14.8 millidegrees in the *θ*-axis. In addition, we determined the thresholds of the X-, Y-, and θ-axes as X_T_ = ±0.14 μm, Y_T_ = ±0.14 μm, and θ_T_ = ±0.4 millidegrees around their corresponding targets.

**Figure 10:**
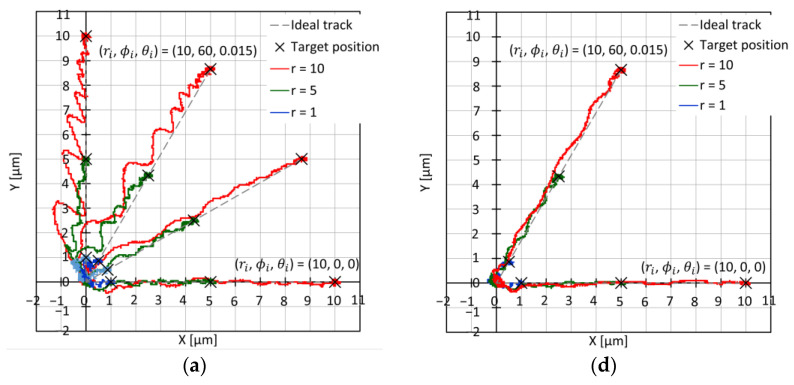

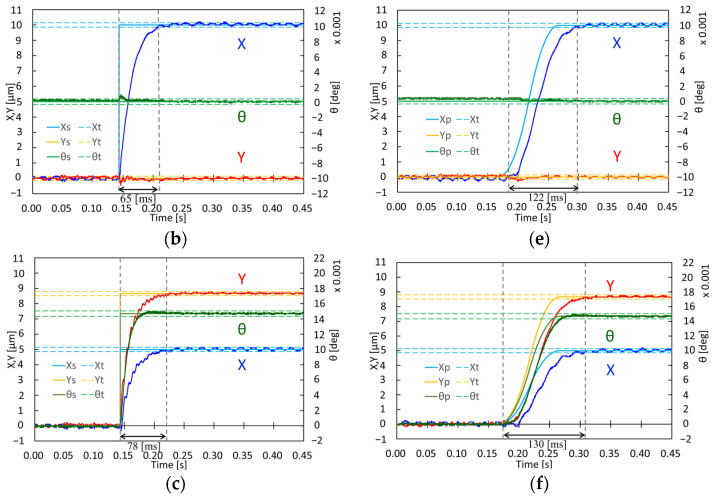
PID control of one step motion (left: step reference, right: parabolic reference with rise time of 100 ms): (**a**) plots of XY trajectories along $\phi_{i}=0,\;30,\;60,\;\mathrm{and}\;90{}^{\circ}$; (**b**) plots of XYθ vs. time for $\left(r_{i},\;\phi_{i},\;\theta_{i}\right)=\left(10\;\mu m,\;0{}^{\circ},\;0\;m{}^{\circ}\right)$; (**c**) plots of XYθ vs. time for $\left(r_{i},\;\phi_{i},\;\theta_{i}\right)=\left(10\;\mu m,\;60{}^{\circ},\;14.8\;m{}^{\circ}\right)$; (**d**) plots of XY trajectories along $\phi_{i}=0\;\mathrm{and}\;60{}^{\circ}$; (**e**) plots of XYθ vs. time for $\left(r_{i},\;\phi_{i},\;\theta_{i}\right)=\left(10\;\mu m,\;0{}^{\circ},\;0\;m{}^{\circ}\right)$; (**f**) plots of XYθ vs. time for $\left(r_{i},\;\phi_{i},\;\theta_{i}\right)=\left(10\;\mu m,\;60{}^{\circ},\;14.8\;m{}^{\circ}\right)$.

Figure 10 shows the experimental results for PID control of a single motion. The step-shaped targets of the X-axis were determined as X_S_, Y_S_, and θ_S_ were similarly defined. As shown in Figure 10a for the XY trajectories, we succeeded in precisely controlling the position of the free leg, although non-negligible oscillations in the X- and Y-axes were generated for the directions of 60° and 90°. Figure 10b,c compares the plots of X, X_S_, Y, Y_S_, θ, and θ_S_ vs. time for the 0 °and 60 °directions, and their settling times were approximately 65 and 78 ms, respectively. We assumed that the oscillation was attributed to electrical noise from a commercial 50 Hz AC power supply, as observed in Figure 10b,c,e,f.

Figure 10d–f shows the experimental results for the parabolic-shaped references with a rise time of 100 ms. Similarly, we define the parabolic-shaped target of the X-axis as X_P_, Y_P_, and θ_P_. Figure 10d shows the XY trajectories. Figure 10e,f shows plots of X, X_P_, Y, Y_P_, θ, and θ_P_ vs. time for the 0° and 60° directions, and their settling times were approximately 122 and 130 ms, respectively. The oscillations decreased to ±0.5 μm in the X and Y axes, as shown in Figure 10.

## 6. Conclusions and Future Prospects

The design and experiments described in this study proved that the realization of a centimeter-sized XYθ position sensor is possible, which is sufficiently compact to attach a centimeter-sized holonomic inchworm robot and can simultaneously and precisely measure XYθ displacements, with a measuring cycle of 0.1 μs, resolution of 0.1 μm and 0.4 millidegrees, measurement accuracy of 0.06–0.19%, and range of 16 × 16 mm^2^ and −25–25°. We demonstrated the sequential positioning control of the multiple-step motion from the centimeter to the micrometer range. We have also demonstrated PID control of the one-step motion from the micrometer to the sub-micrometer range.

In future research, we plan to decrease the noise, obtain the frequency response, improve the pseudo-differential filter, and automatically tune the PID gains. For multiple-step motion, we plan to decrease the threshold around the target position to the sub-micrometer range to achieve more precise positioning control. We also plan to shorten the time required for precise positioning by applying motion compensation. Eliminating cyclic vibrations during the switching of the supporting leg is also an important approach for more accurate control.

For precise one-step motion, we plan to develop a systematic method for obtaining PID gains and investigate more efficient control sequences, such as optimal, model-following, and acceleration feedback control.

Finally, the improvement of the calibration of the XYθ displacement sensor, reduction of the friction between the legs and floor, and reduction of the hysteretic nonlinearity of the PAs are required for more precise and faster positioning.

To expand the applicability of the holonomic inchworm robot, we developed manipulators to be mounted on the robot, which can precisely manipulate minute parts, such as electronic chip components, MEMS, biological cells, microorganisms, and microfossils. We also developed a precise control method for the revolution around the manipulator tip within a narrow microscopic image.

The final goal of this research is to realize the automation of a mobile robotic factory organized by multiple centimeter-sized robots equipped with various tools for the multi-axial processing of biological samples and the assembly of millimeter-sized micro-robots and mechanisms.

## Figures and Tables

**Table 1:** Performance of the inchworm mobile robot.

Characteristic Value	Quantity
Step length (120 V)	~65 μm
Resolution (15–25 °C, less than 50% rH)	Less than 10 nm
DoF	X, Y, θ
Natural Frequency (blocked free)	X: 413, Y: 418, θ: 476 Hz
Maximum Velocity [frequency]	~6.5 mm/s [100 Hz]
Repeatability (CV; ratio of SD of final points to a path length with 10 mm path) [frequency]	~3% [100 Hz]
Maximum payload	<1000 g
Dimension	86 × 86 × 15 mm
Weight	100 g

**Table 2:** Performance of the piezoelectric actuator.

Characteristic Value	Quantity
Displacement (100 V)	95.5 ± 5 μm
Generative Force (100 V)	18.0 N
Spring constant	115,000 N/m
Capacitance	1.04 μF
Resolution (15–25 °C, less than 50% rH)	1.52 nm
Natural Frequency (blocked free)	1.45 kHz
Dimension	12.9 × 6.4 × 9.2 mm
Weight	4 g

**Table 3:** Performance of encoder (TA-200, Technohands).

Characteristic Value	Quantity
Resolution [μm/count]	0.1
Maximum measurement speed [mm/s]	800
Dimension [mm]	15 × 10 × 1.5

**Table 4:** Specifications of the XYθ position sensor.

Characteristic Value	Quantity
Measurement range X × Y [mm], θ [°]	16 × 16, ±25
Measurement resolution in X (Y) [μm], θ [millidegrees]	0.1, 0.3
Uncertainty in static state in X (Y) [μm], θ [millidegrees]	±0.2, ±0.6
Measurement frequency [MHz]	2.86
Maximum measurable speed [mm/s]	800
Principle of measurement	Incremental
Measurement accuracy in X and Y (−8~8mm) [%]	0.08–0.18
Measurement accuracy in θ (−25~25°) [%]	0.06–0.19

## Data Availability

The data that support the findings of this study are available from the corresponding author, O.F., upon reasonable request.

## Supplementary Materials

The following supporting information can be downloaded at: https://www.mdpi.com/article/10.3390/mi14020375/s1; Table S1—Specification and score of the representative micromanipulation robots.; Figure S1—Plots of XYθ-axes errors vs. XYθ-axes simultaneous displacement.; Video S1 3DOF motion of holonomic inchworm robot.
