# Supplementary material for: Precise Position Control of Holonomic Inchworm Robot Using Four Optical Encoders
Source: Micromachines (Basel). 2023 Feb 2;14(2):375. doi: 10.3390/mi14020375 (PMC9967612; doi:10.3390/mi14020375)
Supplement: Supplementary file 1 [file micromachines-14-00375-s001.zip › Supplementary Materials-Table S1 and Figure S1.pdf]

Supporting Information

Precise position control of holonomic inchworm robot using four optical encoders

K. Tanabe, M. Shiota, E. Kusui, Y. Iida, H. Kusama, R. Kinoshita, Y. Tsukui, R. Minegishi, Y. Sunohara, O. Fuchiwaki

Table S1 Specification and score of the representative micromanipulation robots.

Micromanipulations (Score)	Dimension [mm]	DoF	Range [mm, deg]	Velocity [mm/s]	Resolution [nm]	Mass [g]	Payload [g]
Inchworm<br>(20)	86×86×15<br>(3)	XYθ_z	∞×∞×∞<br>(4)	6.5<br>(3)	10<br>(3)	100<br>(4)	1000<br>(3)
USM<br>(20)	55×55×14<br>(4)	XYθ_z	∞×∞×∞<br>(4)	31.5<br>(5)	5<br>(2)	145<br>(3)	300<br>(2)
PER-Hexpod<br>(19)	68×60×59<br>(2)	XYZ<br>$\theta_x\theta_y\theta_z$	∞×∞×0.018<br>×0.05×0.03×∞<br>(5)	1.89<br>(2)	4.53<br>(4)	450<br>(2)	10000<br>(4)
miBot<br>(19)	20.5×21.7<br>×12.5<br>(5)	XYZθ_z	20×20×5.81×∞<br>(2)	1.5<br>(1)	0.02<br>(5)	6<br>(5)	6<br>(1)
XY stage<br>(13)	345×345<br>×110<br>(1)	XY	100×100<br>(1)	10<br>(4)	1000<br>(1)	7600<br>(1)	16000<br>(5)

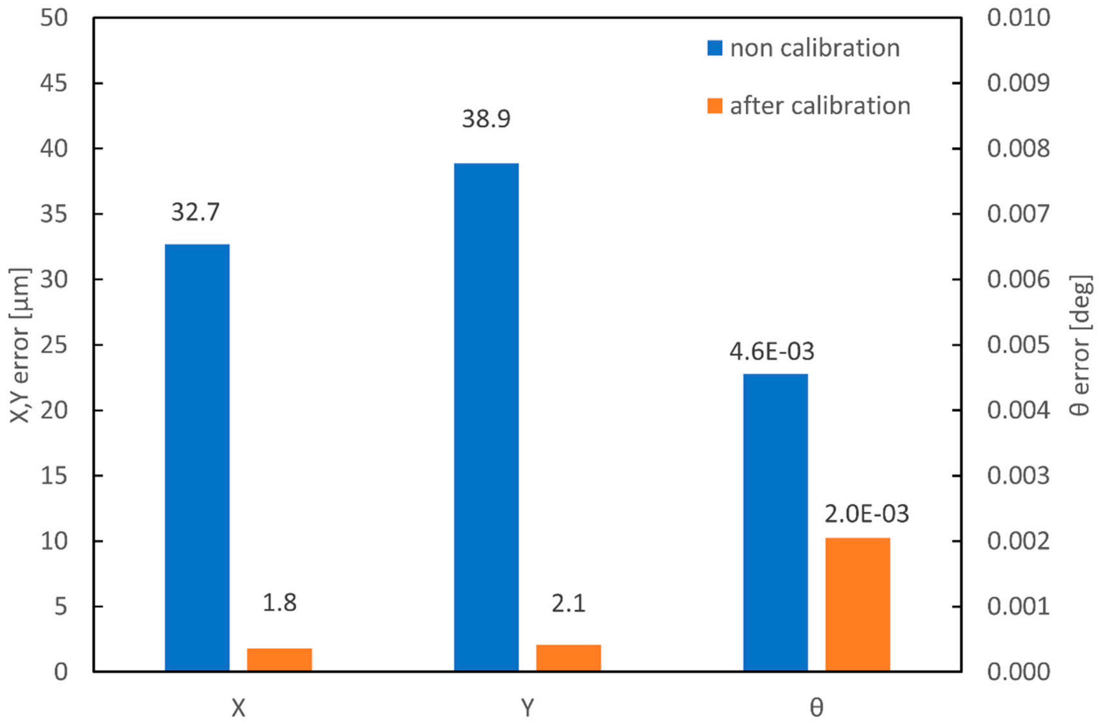

Fig. S1 Plots of XYθ-axes errors vs. XYθ-axes simultaneous displacement for (X, Y, θ) = (4000μm, 4000μm, 5˚)

Movie S1 3DOF motion of holonomic inchworm robot.

See attached movie
